# Investigating stress, anxiety, and coping strategies among higher education students in extreme contexts: insights from Romania during the COVID-19 pandemic

**DOI:** 10.1186/s12889-024-20949-0

**Published:** 2024-12-18

**Authors:** Mihaela Simionescu, Ali B. Mahmoud, Wadim Strielkowski, Nicolae-Marius Jula

**Affiliations:** 1 Faculty of Business and Administration, University of Bucharest, Bucharest, Romania; 2 Institute for Economic Forecasting, Romanian Academy, Bucharest, Romania; 3 Queen Mary University of London, London, UK; 4 St. John’s University, Queens, NY USA; 5 Czech University of Life Sciences Prague, Prague, Czech Republic; 6 University of California, Berkeley, USA

**Keywords:** Extreme contexts, Stress, Anxiety, COVID-19 pandemic, Students, Higher education

## Abstract

**Background:**

Using the perspective of the recent COVID-19 pandemic, which represents a public health challenge that also affects education and the psychological well-being of students, this paper aims to assess the vulnerability to anxiety and stress of the Romanian students from the Faculty of Administration and Business of the University of Bucharest pursuing their degrees in administration and business.

**Methods:**

Our study is based on the results of the survey that was administered to a sample of 422 students (39.6% females and 60.4% males) selected from the pool of 2000 recruited respondents. We employed the ANOVA/linear Dependent Dirichlet Process mixture model to explain the causes of stress and anxiety after various grouping variables represented by gender, specialisation, as well as labour market status.

**Results:**

Our results revealed that more than 80% of the students in the sample yielded medium and high vulnerability to stress, while 64% of the respondents were affected by severe anxiety (high frequency of psychological, social, and physical problems that were difficult for them to control). The most important stressors were the fear of getting infected and social distancing, while self-control was considered the most effective coping strategy by 62.6% of employed students.

**Conclusions:**

These results allow us to provide practical recommendations for effectively coping with stress and anxiety among students in Romania and beyond and to help stakeholders and policymakers design strategies for strengthening students’ resilience, mental health, and well-being in case of future pandemics or other extreme contexts.

**Supplementary Information:**

The online version contains supplementary material available at 10.1186/s12889-024-20949-0.

## Background

The COVID-19 pandemic caused many adverse effects on healthcare, business, economics, and social life [1]. One significant but often underestimated outcome was its profound impact on higher education, disrupting traditional on-campus learning, participation in lectures and seminars, and personal interactions [2, 3]. The social distancing that was a part of lockdowns aimed at restraining the pandemic also resulted in social isolation and online learning for millions of students. This sudden shift led to significant deterioration in the mental health and well-being of many students, resulting in increased anxiety and stress and adversely affecting their academic performance. This study aims to investigate the interrelationships among stress levels, anxiety levels, coping strategies, and demographic characteristics of higher education students in Romania during the COVID-19 pandemic, an example of an extreme context [4–6].

The COVID-19 pandemic had major outcomes for public health worldwide [7–9]. In addition to its health impacts, the pandemic had significant educational and social repercussions, such as the closure of university campuses and the shift to online learning and social distancing worldwide [10–14]. Starting from March 2020, COVID-19 severely disrupted the lives of university students worldwide, and the potential health risks they were facing proved to be even worse (and this is not to mention that the pandemic is far from becoming endemic). The pandemic exacerbated known risk factors for student mental health and other health problems while hurting students’ academic performance and jeopardising their prospects for college admissions. Some studies found that nearly half of currently enrolled students state that COVID-19 is likely or likely to negatively impact their ability to earn a degree or diploma [15, 16].

Thus, the educational disruptions caused by the COVID-19 pandemic led to immense stress and anxiety for students, who are particularly vulnerable due to various factors influencing their mental health. The prevalence of depressive symptoms in university students during this period was notably higher than in other groups and varied significantly among individuals [17]. After the COVID-19 pandemic, students in many countries reported mental health problems (anxiety and/or depression) due to the disruption of their studies, indicating a higher rate of general mental health problems. For example, a survey of university students in the United Kingdom found that 58% of university students said their mental health had worsened due to the COVID-19 pandemic [18]. Another similar survey from the United States with over 190 students also reported the negative impacts of the pandemic on students’ mental health and called for preventive measures [19].

According to another study conducted for the U.K. Parliament [20], the mental health of higher education students was a growing concern even before the COVID-19 pandemic. However, during the COVID-19 pandemic, the number of students experiencing negative emotions and psychological problems increased [21], suggesting a possible impact on mental health. Likewise, a study from China found that about 40% of teenage university female students suffer from depression [22]. All of the above shows that mental issues are notorious for the students and that “black swans”, just like COVID-19, might only deepen those issues and increase the stress and anxiety of the young minds [23].

The primary aim of this study is to explore how the extreme context of the COVID-19 pandemic has affected the stress and anxiety levels of higher education students in Romania. By collecting and analysing student responses about their attitudes, behaviours, and self-reported mental health, this study seeks to understand the interrelationships among these variables.

This study fills a gap in the literature by addressing the mental health implications of extreme contexts, such as the COVID-19 pandemic, for Romanian higher education students. Furthermore, our paper’s main novelty is its attempt to measure the vulnerability to anxiety and stress of Romanian students using robust econometric modelling rather than a qualitative approach. In addition, our paper focuses on stress and anxiety after various grouping variables represented by gender, specialisation, as well as the labour market status predetermined by the studies dealing with the determinants of stress and anxiety [24] as well as several recent similar studies conducted in Romania, the Czech Republic, or Russia [25, 26]. Our results compare the vulnerability to stress and identify the specific stress factors and strategies for Romanian students during the pandemic. The results obtained are discussed, and the practical implications and limitations of the research are drawn. The last section of this paper presents the main results and pathways for further research.

### Literature review

The COVID-19 pandemic has highlighted the vulnerability of students, necessitating immediate attention and support for their mental health and well-being [27–30]. During the COVID-19 pandemic, students’ mental health has been affected to some extent, with an increase in the number of students experiencing negative emotions and psychological problems suggesting a possible impact of COVID-19 on them [31, 32]. According to Khan et al. [33], a survey of Bangladeshi university students during the COVID-19 pandemic yielded that 28.5% of respondents suffered from stress, 33.3% from anxiety, and 46.92% from depression. In addition, Hasan and Bao [34] showed that the transition to e-learning and the fear of missing the school year were associated with higher levels of psychological anxiety for some students. Furthermore, other studies report that student-athletes may have experienced the stress of the COVID-19 pandemic in unique ways, indicating higher levels of depression and anxiety [35–37].

Many studies have reported an increase in symptoms of depression, generalised anxiety disorder, and overall distress among students during the pandemic [38]. A study from Pennsylvania showed that students indicating the negative impact of COVID-19 on their “mental health” reported higher levels of psychological stress across all of its domains [39].

Regarding the subject of anxiety, the results from many studies showed its prevalence among university students during the COVID-19 pandemic. For example, a study by the National Collegiate Athletic Association (NCAA) in the U.S. found that in the spring of 2020, 27% of female student-athletes and 14% of male student-athletes experienced “severe anxiety” following the COVID-19 restrictions [40]. Similar results emerged from another study examining the impact of COVID-19 on U.S. college students, which found an increase in sedentary lifestyles, anxiety, and depressive symptoms [41]. Additionally, in a few studies, clinicians rated anxiety, stress, severe anxiety, depression, academic achievement, social isolation, family, and adjusting to a new environment causing problems in students seeking mental health care due to COVID-19 more often [42–44].

Interestingly, similar to pre-pandemic occurrences, adults in poor general health (reflecting both physical and mental health) reported higher rates of anxiety and/or depression than those in good health during the pandemic. The already high likelihood of comorbid mental disorders was exacerbated by their vulnerability to serious illness caused by the coronavirus [45, 46]. The December 2020 KFF Health Tracking survey also found that families who lost their jobs were more likely to report that anxiety or stress related to the COVID-19 pandemic negatively impacted their mental health.

Another survey conducted in the U.S. in 2020 found that a higher percentage of households that lost their jobs reported that pandemic-related anxiety and stress had at least adverse effects on their mental health and well-being, such as sleep or eating problems, increased alcohol or substance use, and worsening of the chronic diseases [47].

Several studies on undergraduates demonstrated higher rates of anxiety, depression, and financial insecurity due to the COVID-19 pandemic [48–50]. In general terms, the psychological literature predicts an increase in anxiety-related disorders during stressful times such as pandemics due to the prevalence of associated increases in stress, anxiety, and depression, all of which tend to be typical for students [51–53]. Even studies from far apart countries, such as China and Poland, demonstrate that the prevalence of depressive symptoms in university students is not only higher than in other groups but also tends to vary among individuals [54, 55].

Empirical studies from U.S. institutions clearly demonstrate that college students experienced severe mental distress during COVID-19 [56–59], although these studies often had small sample sizes and rarely investigated whether specific groups were more vulnerable than others during the pandemic. In several studies [60], students who reported that they were currently or previously taking medication for mental health issues or who received or attempted to access health care for individuals or family members during the pandemic also tended to experience higher levels of depression and had a lower quality of life. Among the multiple stressors mentioned by these students were their fears and concerns about their health or the health of their loved ones, difficulty concentrating, sleep disturbances, decreased social interaction due to physical distancing, as well as increased anxiety about school performance [61]. According to another study commenced by the City University of New York (CUNY) [62], the reported frequency of these feelings among students has more than doubled, likely due to the current COVID-19 outbreak, which is worrisome given its association with poor education and health outcomes. In addition to the high burnout category, the study reported student depression levels ranging from 29 to 38%, which may indicate an increase in epidemic-related depressive symptoms among university students [62].

### Theoretical underpinnings

The Transactional Theory of Stress and Coping by Lazarus and Folkman [63] provides a robust theoretical framework for studying stress, anxiety, and coping strategies among higher education students in extreme contexts, such as during the COVID-19 pandemic in Romania. This theory suggests that stress results from an individual’s appraisal of a situation and their ability to cope with it [64]. In the context of the COVID-19 pandemic, where students encountered challenges like sudden shifts to online learning, social isolation, and health concerns, understanding how they assessed these stressors and coped with them aligns well with the principles of the Transactional Theory of Stress and Coping [65].

Recent studies have demonstrated the impact of the COVID-19 pandemic on Romanian university students, revealing a significant increase in sedentarism and reduced energy for physical activity among students, which had implications for their psychological well-being [64]. This finding underscores the importance of exploring coping strategies among students, as physical well-being is closely tied to mental well-being, a key aspect considered in Lazarus and Folkman’s Transactional Theory of Stress and Coping [64]. Furthermore, Ionescu et al. [64] highlighted the radical changes in the Romanian education system due to the pandemic, requiring swift adaptation to online learning processes by both teachers and students [64]. This rapid adjustment aligns with Lazarus and Folkman’s theory, which underscores the significance of coping strategies in response to stressors that demand behavioural and cognitive changes [64].

Additionally, Constantinescu et al. [66] evaluated the impact of the pandemic on stress, anxiety, and depression levels among resident doctors specialising in gastroenterology in Romania [64]. Understanding the coping mechanisms utilised by these medical professionals during the crisis offers valuable insights into how individuals in high-stress environments manage their well-being, a central tenet of the Transactional Theory of Stress and Coping [64]. Moreover, Dascalu et al. [67] discussed the prospects of COVID-19 vaccination in Romania, highlighting challenges and potential solutions, emphasising the need for adaptive coping strategies at the societal level to effectively combat the pandemic [64]. This macro-level perspective on coping strategies aligns with the Transactional Theory, which recognises the role of both individual and collective coping mechanisms in addressing stressors [64].

In conclusion, Lazarus and Folkman’s Transactional Theory of Stress and Coping [63] offers a comprehensive framework for understanding how higher education students in extreme contexts, such as during the COVID-19 pandemic in Romania, experience stress, anxiety, and employ coping strategies. By examining how students and other stakeholders in the educational system appraise stressors and utilise coping mechanisms, researchers can gain valuable insights into promoting well-being and resilience in challenging circumstances.

### Data

Our data originates from our own online survey administered among higher education students in Romania to assess their vulnerability to stress and anxiety during the COVID-19 pandemic, which serves as an example of an extreme context. The survey took place in January 2022 using an online questionnaire. Ethical criteria were met via the approval of the faculty ethics committee (ethical approval no. 52/2022 signed by the ethical committee of the University of Bucharest). The students were informed about the scope of this research, and their anonymity and confidentiality were fully ensured. All participants in the study provided electronic informed consent, thereby ensuring that they fully understood the research scope, procedures, and their right to withdraw at any time. Threats related to external and internal validity were not identified, while previous studies confirm construct validity.

In this study, we employed the STROBE cross-sectional reporting guidelines. A link was emailed to students, asking them to complete the questionnaire in Romanian. The survey took about 15 min to complete. Some subjects refused to participate, and the response rate was 80.75%. The questions were selected using the design from Simionescu et al. [26]. Some actions were undertaken to avoid potential non-response bias. Before administering the questionnaire, a meeting with the potential respondents was organised, and the research purpose was presented. Instructions were given to the respondents to ensure that the questions were fully understood. After the collection of the data, missing responses were observed in the case of six students who were taken out of the study. No compensation was provided for participating in the survey.

Therefore, higher education students represent the population analysed in this paper. The population size in the academic year 2021/2022 was 2000 students enrolled in the Bachelor program. The survey was administered to the students on a voluntary basis. A limitation of this research is the fact that the sample was not representative at the national level, but the representativeness was ensured for higher education students from the University of Bucharest, one of the most prestigious universities in Romania. Another limitation is the potential sources of bias caused by the lack of honesty in answering certain questions.

The online questionnaire served as the instrument for collecting the data. It consisted of 28 questions organised as follows: 20 questions used to measure stress scores, one question to identify the most important stressor during the pandemic (fear of virus, fear of vaccine, social distancing, mask wearing, online lectures, overwork for employees), one question to identify the most efficient strategy to cope with the stress during the epidemic (self-control, family support, colleagues, professors and friends support, spiritual support (belief in power of God, prayers etc.)), one questions including 5 sub-questions to measure the anxiety score (the degree in which the following aspects were affected: working capacity, the management of household activities (cleaning, cooking, paying bills, shopping), recreational social activities (visits, walks with other people, etc.), individual recreational activities (reading, gardening, walking alone, etc.), maintaining close relationships with others), one question related to the labour market status (employed/non-employed), one question related to gender (male, female, other), one question related to the living environment (living alone, with parents, with other relatives, by rent or living in the students guesthouse), one question regarding age (19 years old, 20 years old, 21 years old, 22 years old, more than 22 years old), one question related to the specialisation (business administration, public administration, economic cybernetics, marketing, business administration in English). The responses related to anxiety measurement are scaled from 0 to 8, and they correspond to variants like neither (0 points), somehow (2 points), certain (4 points), in a significant way (6 points), very severe (8 points). There are five possible responses for each question related to stress vulnerability: always (1 point), almost always (2 points), neutral (3 points), almost never (4 points), and never (5 points). The questions related to stress evaluation refer to:

- Good health;
- Normal weight;
- Regular physical exercises;
- Sleeping enough at least 4 days/week;
- Balanced meals taken each day;
- Affection received;
- Relatives’ support;
- Smoking less than 10 cigarettes/day;
- Consumption of less than 3 cups of coffee/coke/energising drink;
- Consumption of less than 5 alcoholic drinks/week;
- Involvement in social activities;
- Entertainment activities each week;
- Good support from religion;
- Capacity to communicate the feelings to others;
- Strong network of friends;
- Efficient time management;
- Enough income;
- Quiet break each day;
- Good communication with members of the household/family regarding personal difficulties;
- Confidence in at least one friend.

We employed the Miller and Smith scale to calculate stress scores for the 20 questions since these items sum up the biological, behavioural, psychological, social, and economic characteristics of the individuals in the sample. This scale was previously applied by Simionescu et al. (2022) to evaluate the vulnerability to stress of nursing students from Romania before and during the pandemic. The internal consistency was measured using Cronbach’s alpha, which resulted in a score of 0.83, suggesting good consistency.

The stress and anxiety scores (in points) allow us to establish more categories of stress and anxiety. The intervals are presented below:

- [0;10): weak anxiety;
- [10;20): medium anxiety;
- [20;40]: high anxiety;
- [0;10]: resistance to stress;
- [11;30): weak vulnerability to stress;
- [30;50): medium degree of stress;
- [50;75): high vulnerability to stress;
- [75;80): extreme mood of stress.

Gender was used as a grouping qualitative characteristic. The population is heterogeneous, and the average qualitative characteristic of the population (p) is 0.5. The significance level is 5% (z score = 1.96 for this significance level), and the maximum permissible error limit is ± 5%: $\:{\varDelta\:}_{W}=\pm\:5\text{\%}$.

$$\:n=\frac{{z}^{2}\cdot p\left(1-p\right)}{{\varDelta\:}_{w}^{2}+\frac{{z}^{2}\cdot p\left(1-p\right)}{N}}=\frac{{1.96}^{2} \cdot 0.25}{0.0025+\frac{3.8416 \cdot 0.25}{2000}}=322.26\cong\:323\:students$$

However, more students than planned filled in the questionnaire, and the sample size was 422. Z test was used to check for sample representativeness. 39.6% of the students in the sample are females, while 43% is the share of females in the population. The null hypothesis of the z-test states equal percentages for females in population (p) and sample (w).

$$\:{z}_{calculated}=\frac{w-p}{\sqrt{\frac{p \cdot (1-p)}{n}}}=\frac{39.6\%-43\%}{\sqrt{\frac{43\% \cdot 57\%}{422}}}=\frac{-3.4\%}{\sqrt{\frac{0.2451}{422}}}=-1.410794$$

In this case, $\:\left|{z}_{calculated}\right|<1.96,$ and the null hypothesis is not rejected, which suggests that the sample is representative of the population according to gender.

## Materials and methods

For our empirical model, we employed the ANOVA/linear Dependent Dirichlet Process (DDP) mixture model to observe how various characteristics explain stress scores and anxiety scores. We employed the methodology proposed by Karabatsos and Walker (2012). Let us consider the variables $\:X={\left(\left(1,{x}_{i}^{T}\right)\right)}_{nx(p+1)}$ and $\:y={({y}_{1},\dots\:,{y}_{n})}^{T}$. In this case, i=1,…,n is used as an index for students in the sample. For one constant (1) and p covariates, $\:x={(1,{x}_{1},\dots\:,{x}_{p})}^{T}$, the regression parameters are denoted by $\:\beta\:={({\beta\:}_{0},{\beta\:}_{1},\dots\:,{\beta\:}_{p})}^{T}$, where $\:{\beta\:}_{0}$ is the intercept while $\:{\beta\:}_{1},\dots\:,{\beta\:}_{p}$ represent the slopes associated with covariates. $\:{\sigma\:}^{2}$ are the errors ($\:{\epsilon\:}_{i})$ variance.

The normal distribution for the parameters $\:\mu\:$ and $\:{\sigma\:}^{2}$ is demoted by $\:N\left(\mu\:,{\sigma\:}^{2}\right).$ Normal p.d.f. is represented by $\:n\left(y|\mu\:,{\sigma\:}^{2}\right)=\frac{1}{\sigma\:\sqrt{2\pi\:}}\text{e}\text{x}\text{p}(-\frac{{(y-\mu\:)}^{2}}{2{\sigma\:}^{2}})$. The likelihood function of y given x with parameters $\:\vartheta\:=(\beta\:,{\sigma\:}^{2})$ is represented as $\:f\left({y}_{i}|x;\vartheta\:\right).$

Let us start with a linear model:

$$\:{y}_{i}={x}_{i}^{T}\beta\:+{\epsilon\:}_{i},\:\:{\epsilon\:}_{i}\to\:N\left(0,{\sigma\:}^{2}\right),\:i=\text{1,2},\dots\:,n$$

Within this context:

$$\:f\left({y}_{i}|{x}_{i};\vartheta\:\right)=n\left({y}_{i}|{x}_{i}^{T}\beta\:,{\sigma\:}^{2}\right),\:i=\text{1,2},\dots\:,n$$

OLS estimates are computed as: $\:\widehat{\beta\:}={\left({X}^{T}X\right)}^{-1}{X}^{T}y,\:{\widehat{\sigma\:}}^{2}=\frac{1}{n-p-1}\sum\:_{i=1}^{n}{({y}_{i}-{x}_{i}^{T}\widehat{\beta\:})}^{2}$, $\:y={({y}_{1},\dots\:,{y}_{n})}^{T}$ and $\:X={\left(\left(1,{x}_{i}^{T}\right)\right)}_{n*(p+1)}$

The general form of non-parametric model (BNP) is given by:

$$\:f\left(y|x;\:\vartheta\:\right)=\int\:f\left(y|x,\tau\:,\theta\:\right)d{G}_{x}\left(\theta\:\right)=\sum\:_{j=1}^{\infty\:}f\left(y\right|x,\tau\:,{\theta\:}_{j}\left(x\right){\omega\:}_{j}\left(x\right)$$

$\:\left\{f(.|x,\tau\:,\theta\:)\right\}:(\theta\:,\tau\:)\}\in\:{\Theta\:}$ represents kernel densities

$\:{\omega\:}_{j}\left(x\right)$ is mixing weights of unitary sum for any $x\in\varkappa$

$\:{\delta\:}_{\theta\:\left(x\right)}(.)$ probability measure which degenerates at $\:\theta\:\left(x\right)$

$\:\tau\:$- other coefficients that do not belong to the mixture

$\:{\left\{{\omega\:}_{j}\left(x\right)\:\right\}}_{j},\:{\left\{{\theta\:}_{j}\left(x\right)\:\right\}}_{j}$ infinite collections of processes indexed after $\varkappa$

The prior distribution corresponding to coefficients of the Bayesian density regression model:

$$\:\vartheta\:=(\tau\:,{\left({\omega\:}_{j}\left(x\right),{\theta\:}_{j}\left(x\right)\right)}_{j}),\:x\in\:\:\varkappa$$

Dependent Dirichlet Process (denoted by DDP) is employed by many Bayesian density regressions. DDP prior is represented as $G_x\sim DDP\left(\alpha,\;G_{0x}\right)$. The random distribution is written as $\:{G}_{x}=\sum\:_{j=1}^{\infty\:}{\omega\:}_{j}\left(x\right){\delta\:}_{{\theta\:}_{j}\left(x\right)}(.)$. The stick-breaking weights are computed as:

$$\:{\omega\:}_{j}\left(x\right)={v}_{j}\left(x\right)\prod\:_{l=1}^{j-1}\left(1-{v}_{j}\left(x\right)\right),\:j=\text{1,2},\dots\:$$

$$\:{v}_{j}\sim{Q}_{j},\:{v}_{j}:\varkappa\to\:[01,]$$

$$\:{\theta\:}_{j}\left(x\right)\sim{ind\:G}_{0x}$$ (the atoms)

ANOVA/linear DDP model is based on the following mixing distribution:

$$G\;Stick-Breaking\,((a_j,b_j)_j,G_0)\,<=>\,G_X(\theta)\sim ANOVA-DDP\,((a_j,b_j)_j,G_0)$$

$$\:{G}_{x}\left(\theta\:\right)=\sum\:_{j=1}^{\infty\:}{\omega\:}_{j}\left(x\right){\delta\:}_{{\theta\:}_{j}\left(x\right)}\left(\theta\:\right)$$

$$\:{\theta\:}_{j}\left(x\right)={x}^{T}{\beta\:}_{j}$$

$$\:{\beta\:}_{j}|\mu\:$$

$$\:T\sim{iid\:G}_{0}=N(\mu\:,T)$$

Normal kernel $$\:n\left(y\right|\theta\:,{\sigma\:}^{2})$$

In this paper, grouping variables are represented by gender, specialisation, and labour status of the students.

$$\:{{(y}_{i\left(h\right)})}_{i\left(h\right)}^{{n}_{h}}|{X}_{h}\sim f\left({y}_{h}|{X}_{h}\right),\:h=1,\dots\:,{N}_{h}$$

$$\:f\left({y}_{h}|{X}_{h}\right)=\sum\:_{j=1}^{\infty\:}\left\{\prod\:_{i\left(h\right)=1}^{{n}_{h}}n\left({y}_{i\left(h\right)}\right|{x}_{i\left(h\right)}^{T}{\beta\:}_{j},{\sigma\:}^{2})\right\}{\omega\:}_{j}$$

$$\:{\omega\:}_{j}={v}_{j}\prod\:_{l=1}^{j-1}(1-{v}_{l})$$

$$\:{v}_{j}|\alpha\:\sim Be(1-a,b+aj)$$

$$\:{\sigma\:}^{2}\sim IG(\frac{{a}_{o}}{2},\frac{{a}_{o}}{2})$$

$$\:{\beta\:}_{j}|\mu\:,T\sim N(\mu\:,T)$$

$$\:\mu\:,T\sim N\left(\mu\:|0,{r}_{0}{I}_{p+1}\right)IW\left(T\right|p+3,{s}_{0}{I}_{p+1})$$

A weight equalled with 1 is assigned to each observation. We will consider 3600 Monte Carlo samples from 20,000 generated samples (aside from the selected burnout of 2000 samples).

### Main results

Table 1 presents the distribution of students according to various characteristics. More than 80% of the students in the sample yielded medium and high vulnerability to stress. In comparison, 64% of the individuals reported severe anxiety (this state can be described as worrying too much, which interferes with one’s studies, work, interpersonal relationships, and other parts of life, and is hard to control – the students self-reported psychological, social, and physical problems in their survey questionnaires which is how the severe anxiety is often classified and described in similar studies [57, 68, 69]. Fear of the virus and social distancing were among the most mentioned factors of stress for students: 27.7% and 24.6% of the individuals mentioned these factors. More than half of the students in the sample were employed, and 62.6% considered self-control the most important strategy for coping with stress during the pandemic. Almost 40% of our respondents were 20 years old. Over 60% of the students were males, and 35.4% of the respondents were students in public administration, followed by such fields of study as economic cybernetics (31.3%) and business administration (24.2%).

**Table 1:** Distribution of students according to various variables (relative frequencies in %)

**Category of stress**	**Category of anxiety**	**Factor of stress**	**Strategy**	**Labour status**	**Living environment**	**Age**	**Gender**	**Specialisation**
resistance to stress:	low anxiety: 10%	fear of vaccine: 7.6%	spiritual support: 10.7%	employed: 64.5%	living alone: 7.3%	19 years old: 28.2%	female: 39.6%	business administration: 24.2%
weak vulnerability to stress: 3.3%	medium anxiety: 26.1%	fear of virus: 27.7%	self-control: 62.6%	non-employed: 35.5%	living by rent: 64.7%	20 years old: 39.6%	male: 60.4%	public administration: 35.4%
medium vulnerability to stress: 40.8%	severe anxiety: 64%	social distancing: 24.6%	friends, colleagues, professors support: 11.4%		living with parents: 15.6%	21 years old: 19.9%		economic cybernetics: 31.3%
high vulnerability to stress: 45.3%		mask wearing: 8.8%	family support: 15.4%		living with other relatives: 5.9%	more than 22 years old:12.3%		marketing: 7.1%
extreme vulnerability to stress: 10.7%		online lectures: 19.4%			living at student guesthouse: 6.4%			business administration in English: 2.1%
		overwork for employees: 11.8%						

Source: own calculations

The association between stress scores, anxiety scores, stress factors, and coping strategies and various economic and demographic characteristics (labour market status, age, environment, gender, specialisation) was analysed using the chi-square test (Table 2).

**Table 2:** The association between factors of stress, strategy for coping with stress, anxiety/stress scores and various variables

**Variable **	**Stress scores**	**Anxiety scores**	**Labour market status**	**Environment **	**Age **	**Gender **	**Specialisation **
Factors of stress	14.193 (0.511)	9.207 (0.513)	67.628 (<0.01)	23.632 (0.259)	39.261* (0.001)	11.469* (0.043)	31.729 (0.166)
Strategy for coping with stress	16.236** (0.062)	14.714* (0.023)	2.077 (0.557)	19.193** (0.084)	14.986** (0.091)	9.429* (0.024)	23.541** (0.073)
Stress scores	-	38.014* (<0.01)	16.138* (0.001)	19.457** (0.078)	25.543* (0.002)	4.652 (0.199)	29.935* (0.012)
Anxiety scores	38.014* (<0.01)	-	2.101 (0.350)	10.671 (0.221)	4.715 (0.581)	0.249 (0.883)	12.460 (0.255)

chi-square statistics with *p*-values in brackets, *suggests significance at 5% level, **shows significance at 10%

Source: own calculations

The results in Table 2 suggest that factors of stress during the pandemic were associated with students’ gender and age at a 5% significance level. A significant association was observed between strategies for coping with stress, as well as the stress and anxiety scores, specialisation, age, gender, and environment. Stress and anxiety scores were strongly correlated, while the level of stress was associated with labour status, specialisation, age, and the living environment.

A deeper statistical analysis allows us to provide more insights into these associations, as Appendix 1 suggests. 31.1% of students 19 years old considered online lectures the strongest factor of stress, while 30.8% of people more than 22 years old claimed that social distancing was the most stressful situation in the pandemic. 29.3% of the females mentioned that social distancing was the most important issue for them. 31% of the males were more afraid of the possibility of contracting the COVID-19 virus.

In total, 58.1% of the students living alone, 60.1% of those living by rent, 75.8% of the students living with parents, 72% of those living with other relatives, and 51.9% of students in guesthouses considered self-control to be the best strategy for coping with stress during the pandemic. More than half of the students in each category of age, each category of stress, and each category of anxiety, 68.3% of the females, 58.8% of the males, more than half of the students from business administration, public administration, and economic cybernetics all unanimously indicated self-control as the most efficient strategy for coping with stress.

Students with high or extreme vulnerability to stress also reported high anxiety. For example, 74.9% of the students with high vulnerability to stress also tended to have high anxiety, while 64.4% of those with extreme levels of stress presented high anxiety.

It appears that students who have a job are more stressed than those who do not work during their studies. 46.7% of the non-employed students present vulnerability to stress, while 48.7% of the employed respondents report high levels of stress.

All students from the Business administration programme conducted in English present high vulnerability to stress, while more than 40% of those from business administration, public administration, economic cybernetics, and marketing belong to the category of people with a high level of stress. More than 40% of the students in each age category present high vulnerability to stress.

 Before the Bayesian analysis, the indicators were normalised. (See tables 3, 4 and 5 in Supplementary Material) report the marginal posterior distribution for the intraclass correlation coefficient denoted by ICC, which indicates the proportion of variation in the scores for stress/anxiety caused by heterogeneity between groups. On average, 75.2% of the variation in stress scores is due to heterogeneity between males and females, while this type of heterogeneity causes 81.5% of the variation in anxiety scores. (See table 3 in Supplementary Material) also presents the marginal posterior distributions of the reliabilities of the estimates of the random intercepts as well as random slope parameters over groups.

The 75% posterior intervals for the entire sample indicate the significance of age, labour status, environment, and specialisation for stress scores and the significance of environment and age for anxiety scores. All these factors represent the significant causes for stress levels according to gender, while only age and environment are causes for anxiety levels since the 75% posterior intervals exclude zero only for two covariates in the case of anxiety. If each group is analysed separately, only labour status, environment and specialisation are not significant causes of stress for males.

On average, 79.9% of the variation in stress scores is due to heterogeneity between employed and non-employed students, while this heterogeneity causes 76.9% of the variation in anxiety scores. If the entire sample is analysed, age, specialisation, gender, and environment are significant causes only for anxiety. At the same time, separate analyses for the two groups (employed and non-employed students) revealed that all these factors become the causes of stress and anxiety (see Table 3 in Supplementary Material).

On average, 78.6% of the variation in stress scores is due to heterogeneity between specialisations, while this heterogeneity causes 73.2% of the variation in anxiety scores. If the entire sample is analysed, age, specialisation, and environment are significant causes of stress, while none of the factors represent the causes of anxiety (see Table 4 in Supplementary Material).

The group analysis revealed that labour status, environment, and age are causes for stress and anxiety, while gender is cause only for anxiety (see Table 5 in Supplementary Material).

## Discussion

In general terms, our results resonate with the studies assessing the factors causing or leading to stress, anxiety, and depression among university students worldwide before and after the COVID-19 pandemic. Our research reveals that anxiety and stress related to the COVID-19 pandemic are now included in the social, academic, and physical loads during college years, which is crucial from a psychological developmental perspective, as well as for a person’s mental health. One quite significant cause may relate to the information deluge, particularly misinformation that was disseminated via mostly social media platforms during the pandemic, to which students without a medical condition are more susceptible compared with students with medical conditions. This could be because respondents with greater exposure to COVID-19 would have greater feelings of control over the pandemic and, therefore, would not feel sufficiently threatened for being afraid too much. Furthermore, when comparing the national statistics, it becomes evident that the incidence of mental health problems was substantially higher during the pandemic [70].

A plethora of studies from different countries measured depression, anxiety, and stress levels in university or college students in such places as Canada [71], South Asia [72, 73], or Egypt [74]. Similarly, studies from Spain and Costa Rica [75] and the United States [76] reported elevated levels of stress, anxiety, and depression among university students during periods of confinement and virtual learning. However, none of these studies focused on the depression during the pandemic-induced lockdowns. One notable contribution is that female college students scored higher on measures of depression, stress, and anxiety compared to male students, and this was also true prior to the outbreak (e.g. according to a study from South Korea [77]. This gender difference was also observed in the study by Gijón Puerta et al. [75], where women had higher levels of anxiety than men, and younger individuals reported higher stress and anxiety levels. Our findings are similar to those from another study conducted in the United States that found an increase in changes in physical activity associated with decreased fear about COVID-19, post-traumatic stress disorder, and other mental health outcomes as a consequence of the pandemic [78]. Moreover, Ulrich et al. [76] found high levels of stress, worry, anxiety, and poor sleep among U.S. college and university students during the early months of the pandemic, emphasising the need for universities to provide resources for healthy coping mechanisms.

The role of social support in mitigating stress and anxiety is highlighted in the study by Acoba [79], which found that perceived stress mediates the relationship between social support and mental health outcomes. This aligns with our findings, as self-control was considered the most effective coping strategy by 62.6% of employed students, suggesting the importance of personal and social resources in managing stress. Additionally, the impact of technology and maladaptive coping strategies on student stress levels, as discussed by Lizarte Simón et al. [80], points to the need to consider factors such as smartphone addiction and cyberloafing in future research.

Moreover, our results resonate with the studies mentioned above as well as with some recent studies that employed similar methods [25] or specifically focused on the case of Romania [26, 81]. These consistencies across studies in different contexts support the generalizability of the findings in understanding stress and coping among university students during the pandemic.

### Theoretical contributions

Our study contributes to Lazarus and Folkman’s Transactional Theory of Stress and Coping [63] by demonstrating its applicability in understanding students’ stress and anxiety during an extreme context like the COVID-19 pandemic. The findings expand the theory by highlighting the role of contextual factors, such as the fear of infection and social distancing, in shaping students’ stress appraisals. Furthermore, the predominance of self-control as a coping strategy suggests that the pandemic context may have altered the relative efficacy of different coping mechanisms proposed by the theory.

### Practical implications

Our results highlight the need for stakeholders and policymakers to focus on providing timely and updated information targeted at university students, as this measure can improve awareness about the disease, counteract the epidemic, and decrease fear, which, in turn, would lead to diminishing anxiety and stress. Providing targeted and timely interventions that educate, address the knowledge gap on COVID-19, and fight against information denial would contribute to mental well-being amongst university students, not only during the current pandemic but in future pandemics, and not only in Romania but also across the world. In case of future events similar to COVID-19, it would be important for relevant stakeholders to create and sustain healthy environments for lecturers, students, and staff. The experience with COVID-19 showed that social distancing and lockdowns need not automatically mean social isolation. In fact, the pandemic has shown an array of tools for keeping effective two-way connections and communication, providing social interactions beyond the university curriculum, and spreading information wherever needed.

### Limitations and future research directions

We must acknowledge the limitations of our study stemming from the non-random sample of students and the self-reporting nature of the survey administered to our respondents (not to mention that all the respondents originated from one university in Romania, albeit one of the most prestigious ones). In addition, all students from our sample originated from the bachelor’s degree courses, which limited us to the younger people, sometimes first-year students who had just enrolled on their study programme and did not have much experience with how to function outside their parents’ homes and their familiar environments. However, despite these limitations, our study provides valuable insights into university students’ stress and coping processes during an extreme context, contributing to the theoretical understanding of these phenomena.

When it comes to pathways for further research, in addition to investigating the underlying relationships across different extreme contexts, it also appears interesting to consider other factors that might cause stress and anxiety, such as trust in anti-COVID vaccines, the increased usage of online communication tools and technologies, or the impacts of social networks. Other family-related factors might also be considered, as quantitative studies such as ours can be complemented with qualitative questionnaires where students record their narratives and personal experiences with stress and anxiety during the pandemic. Additionally, it would be interesting to conduct a series of similar studies in several countries apart from Romania and compare the results, which would be especially interesting for groups of technologically more advanced countries, for instance, those with higher Internet penetration and those not as advanced in information technology.

## Conclusions

Our results revealed that the majority (more than 80%) of the students in our sample of Romanian students self-reported medium and high vulnerability to stress, while more than half (64%) of the respondents reported experiencing severe anxiety. Furthermore, the important stressors proved to be the fear of getting infected with a virus and social distancing, while the best strategy to cope with the stress and anxiety was self-control. These results are in accord with other similar studies conducted with university students around the world and mapping the impacts of the COVID-19 pandemic on the mental health of young people.

Our study contributes to Lazarus and Folkman’s Transactional Theory of Stress and Coping [63] by demonstrating its applicability in understanding students’ stress and anxiety during an extreme context like the COVID-19 pandemic. The findings expand the theory by highlighting the role of contextual factors, such as the fear of infection and social distancing, in shaping students’ stress appraisals. Furthermore, the predominance of self-control as a coping strategy suggests that the pandemic context may have altered the relative efficacy of different coping mechanisms proposed by the theory.

In conclusion, our study supports the utility of Lazarus and Folkman’s Transactional Theory of Stress and Coping in understanding students’ experiences during the COVID-19 pandemic. The findings highlight the need for stakeholders and policymakers to consider the role of contextual factors and individual coping strategies in designing interventions to support student mental health during extreme events. Future research could further investigate how the pandemic has modified the stress appraisal and coping processes proposed by the theory across different contexts and populations.

## Supplementary Information

Supplementary Material 1.

## Data Availability

Available on request.

## Abbreviation

COVID-19: Coronavirus pandemic
