# Supplementary material for: Investigating stress, anxiety, and coping strategies among higher education students in extreme contexts: insights from Romania during the COVID-19 pandemic
Source: BMC Public Health. 2024 Dec 18;24:3497. doi: 10.1186/s12889-024-20949-0 (PMC11657389; doi:10.1186/s12889-024-20949-0)
Supplement: Supplementary file 1 — Supplementary Material 1. [file 12889_2024_20949_MOESM1_ESM.docx]

**Appendices**

**Appendix 1**

Express the frequency of the acts reflected by the following 20 sentences (aways, almost always, neutral, almost never, never):

1. I eat at least one balanced meal a day.

2. I sleep 7-8 hours/night at least 4 times / week.

3. I receive and give affection regularly.

4. I have at least one relative I can count on and who lives less than 80 km away.

5. I do exercise at least 2 times/week.

6. I smoke less than half a pack of cigarettes/day.

7. I drink less than 5 alcoholic drinks/week.

8. My body weight is adequate for my height.

9. My income is adequate for basic expenses.

10. I find my strength in my religious faith.

11. I am regularly involved in social activities.

12. I have a network of friends and acquaintances.

13. I have one / more friends I trust.

14. I am in good health (including sight, hearing, teeth).

15. I am able to talk openly about my feelings when I am upset or worried.

16. I usually talk to the family/people I live with about household issues (household activities, money, daily chores).

17. I do something funny at least once a week.

18. I am able to organize my time efficiently.

19. I drink less than 3 cups of coffee or cola / day.

20. I take a quiet break during the day.

21. What is your gender?

a) female

b) male

c) else.

22. What is your age?

a) 19 years old;

b) 20 years old;

c) 21 years old;

d) 22 years old;

e) more than 22 years old.

23. Your specialization is:

a) business administration;

b) public administration;

c) economic cybernetics;

d) marketing;

e) business administration in English.

24. The status on labour market:

a) employed;

b) non-employed.

25. The most efficient strategy to cope with the stress during the epidemic was:

a) self-control;

b) family support;

c) colleagues, professors and friends support;

d) spiritual support (belief in power of God, prayers etc.).

26. The most important stressor during the pandemic was:

a) fear of virus;

b) fear of vaccine;

c) social distancing;

d) mask wearing;

e) online lectures;

f) overwork for employees.

27. Express the degree in which the following aspects were affected (neither, somehow, certain, in a significant way, very severe):

a) working capacity;

b) the management of household activities (cleaning, cooking, paying bills, shopping);

c) recreational social activities (visits, walks with other people, etc.);

d) individual recreational activities (reading, gardening, walking alone, etc.);

e) maintaining close relationships with others.

28. Living environment:

a) living alone;

b) living with parents;

c) living with other relatives;

d) living by rent;

e) living in the students guesthouse.

**Appendix 2**

Posterior Summary Estimates for explaining stress and anxiety scores according to gender

| Parameter | Stress | | | | | Anxiety | | | | |
| --- | --- | --- | --- | --- | --- | --- | --- | --- | --- | --- |
|  | Mean | Standard deviation | 25% | 75% | CUSUM | Mean | Standard deviation | 25% | 75% | CUSUM |
| $\beta_{0}(sample)$ | 42.137 | 32.658 | 16.661 | 63.183 | 0.002 | 33.098 | 14.111 | 33.155 | 40.252 | 0.395 |
| $\beta\left( sample \right):employed$ | 8.326 | 6.793 | 3.897 | 12.513 | 0.003 | -1.018 | 3.282 | -1.883 | 0.989 | 0.38 |
| $\beta\left( sample \right):environment$ | -0.611 | 2.159 | -0.877 | -0.877 | 0.041 | -0.838 | 2.264 | -1.297 | -0.250 | 0.457 |
| $\beta\left( sample \right):age$ | 0.271 | 2.246 | 0.379 | 0.379 | 0.04 | -2.835 | 3.299 | -5.285 | -0.999 | 0.494 |
| $\beta\left( sample \right):specialization$ | 1.021 | 2.327 | 1.610 | 1.610 | 0.04 | -0.250 | 2.097 | -1.030 | 0.248 | 0.431 |
| $\sigma_{sample}^{2}$ | 204.959 | 146.370 | 1.785 | 310.337 | 0 | 72.803 | 31.606 | 77.506 | 78.788 | 0.287 |
| $\mu_{\beta_{0}}$ | 1.845 | 3.255 | -0.400 | 4.137 | 0.489 | 3.427 | 3.266 | 1.268 | 5.563 | 0.491 |
| $\mu_{employed}$ | 0.342 | 3.169 | -1.826 | 2.477 | 0.328 | -0.265 | 2.963 | -2.234 | 1.692 | 0.255 |
| $\mu_{environment}$ | 0.033 | 3.052 | -2.044 | 2.104 | 0.317 | 0.074 | 3.074 | -2.064 | 2.165 | 0.258 |
| $\mu_{age}$ | 0.049 | 3.184 | -2.063 | 2.255 | 0.314 | 0.231 | 3.075 | -1.782 | 2.213 | 0.321 |
| $\mu_{specialization}$ | 0.062 | 3.153 | -2.036 | 2.116 | 0.32 | 0.123 | 3.055 | -1.925 | 2.226 | 0.257 |
| $\tau_{\beta_{0}}$ | 631.560 | 456.725 | 367.313 | 746.070 | 0.519 | 383.340 | 245.142 | 230.169 | 461.570 | 0.492 |
| $\tau_{\beta_{0}: \beta_{employed}}$ | 123.7 | 97.133 | 67.478 | 147.690 | 0.477 | -7.555 | 39.904 | -25.085 | 10.861 | 0.277 |
| $\tau_{\beta_{0}: \beta_{environment}}$ | -8.865 | 42.411 | -26.272 | 10.692 | 0.343 | -6.109 | 39.132 | -24.493 | 14.256 | 0.257 |
| $\tau_{\beta_{0}: \beta_{age}}$ | -4.846 | 43.829 | -22.767 | 14.424 | 0.314 | -33.541 | 43.814 | -51.089 | -8.721 | 0.349 |
| $\tau_{\beta_{0}: \beta_{age}}$ | -17.703 | 45.393 | -32.972 | 4.728 | 0.336 | -15.719 | 41.293 | -32.692 | 6.278 | 0.259 |
| $\tau_{\beta_{employed}}$ | 27.685 | 24.895 | 13.245 | 34.204 | 0.413 | 4.630 | 5.721 | 1.682 | 5.269 | 0.409 |
| $\tau_{\beta_{employed}:\beta_{environment}}$ | -1.695 | 8.746 | -4.919 | 2.002 | 0.339 | 0.358 | 3.775 | -0.794 | 1.372 | 0.387 |
| $\tau_{\beta_{employed}:\beta_{age}}$ | -0.956 | 9.096 | -4.334 | 2.722 | 0.316 | 1.358 | 4.558 | -0.497 | 2.837 | 0.365 |
| $\tau_{\beta_{employed}:\beta_{specialization}}$ | -3.399 | 8.895 | -6.383 | 0.966 | 0.337 | -0.252 | 4.049 | -1.453 | 0.887 | 0.369 |
| $\tau_{\beta_{environment}}$ | 3.345 | 4.038 | 1.417 | 3.835 | 0.464 | 4.476 | 5.348 | 1.622 | 5.237 | 0.4 |
| $\tau_{\beta_{environment}:\beta_{age}}$ | 0.056 | 2.915 | -0.727 | 0.783 | 0.436 | 1.196 | 4.960 | -0.679 | 2.666 | 0.359 |
| $\tau_{\beta_{environment}:\beta_{specialization}}$ | 0.215 | 2.747 | -0.704 | 0.961 | 0.447 | -0.186 | 4.049 | -1.403 | 1.080 | 0.38 |
| $\tau_{\beta_{age}}$ | 3.403 | 3.911 | 1.417 | 3.953 | 0.422 | 8.512 | 9.383 | 3.227 | 10.268 | 0.378 |
| $\tau_{\beta_{age}:\beta_{specialization}}$ | 0.088 | 2.802 | -0.736 | 0.896 | 0.440 | 0.737 | 5.328 | -1.442 | 2.338 | 0.328 |
| $\tau_{\beta_{specialization}}$ | 3.838 | 4.854 | 1.485 | 4.210 | 0.487 | 5.319 | 6.655 | 1.825 | 6.272 | 0.366 |
| $\alpha$ | 2.209 | 2.377 | 0.612 | 2.983 | 0 | 3.561 | 2.670 | 1.682 | 4.699 | 0.494 |
| $\beta_{0}(gender=male)$ | 63.183 | 0.000 | 63.183 | 63.183 | 0 | 39.526 | 1.962 | 37.285 | 41.245 | 0 |
| $\beta_{0}(gender=female)$ | 63.183 | 0.000 | 63.183 | 63.183 | 0 | 40.266 | 0.018 | 40.252 | 40.284 | 0.001 |
| $\beta_{employed}(gender=male)$ | 12.513 | 0.000 | 12.513 | 12.513 | 0 | -0.155 | 1.516 | -1.883 | 1.173 | 0 |
| $\beta_{employed}(gender=female)$ | 12.513 | 0.000 | 12.513 | 12.513 | 0 | -1.868 | 0.020 | -1.881 | -1.862 | 0.001 |
| $\beta_{environment}(gender=male)$ | -0.877 | 0.000 | -0.877 | -0.877 | 0 | 0.204 | 0.504 | -0.371 | 0.645 | 0 |
| $\beta_{environment}(gender=female)$ | -0.877 | 0.000 | -0.877 | -0.877 | 0 | -1.290 | 0.014 | -1.297 | -1.285 | 0.003 |
| $\beta_{age}(gender=male)$ | -0.379 | 0.000 | -0.379 | -0.379 | 0 | -0.783 | 0.196 | -0.999 | -0.612 | 0 |
| $\beta_{age}(gender=female)$ | -0.379 | 0.000 | -0.379 | -0.379 | 0 | -5.231 | 0.084 | -5.285 | -5.206 | 0.001 |
| $\beta_{specialization}(gender=male)$ | -1.610 | 0.000 | -1.610 | -1.610 | 0 | -1.914 | 1.893 | -3.573 | 0.246 | 0 |
| $\beta_{specialization}(gender=female)$ | -1.610 | 0.000 | -1.610 | -1.610 | 0 | -0.798 | 0.020 | -0.812 | -0.786 | 0 |
| $s^{2}(gender=male)$ | 310.337 | 0.000 | 310.337 | 310.337 | 0 | 74.753 | 4.073 | 71.217 | 78.788 | 0 |
| $s^{2}(gender=female)$ | 310.337 | 0.000 | 310.337 | 310.337 | 0 | 92.732 | 22.127 | 77.506 | 102.858 | 0 |
| ICC | 0.752 | 0.200 | 0.587 | 0.996 | 0.511 | 0.815 | 0.109 | 0.747 | 0.883 | 0.496 |
| Reliability ($\beta_{R_{0}})$ | 0.998 | 0.002 | 0.997 | 1.000 | 0.511 | 0.999 | 0.001 | 0.998 | 0.999 | 0.496 |
| Reliability ($\beta_{R_{employed}})$ | 0.945 | 0.064 | 0.918 | 1.000 | 0.481 | 0.880 | 0.095 | 0.824 | 0.953 | 0.438 |
| Reliability ($\beta_{R_{environment}})$ | 0.737 | 0.225 | 0.551 | 0.995 | 0.49 | 0.875 | 0.097 | 0.815 | 0.952 | 0.403 |
| Reliability ($\beta_{R_{age}})$ | 0.739 | 0.223 | 0.546 | 0.995 | 0.469 | 0.928 | 0.061 | 0.895 | 0.973 | 0.391 |
| Reliability ($\beta_{R_{specialization}})$ | 0.749 | 0.220 | 0.564 | 0.996 | 0.467 | 0.887 | 0.094 | 0.835 | 0.962 | 0.397 |
| $\beta_{s^{2}}(acceptance rate)$ | 0.002 | 0.041 | 0.000 | 0.000 | 0 | 0.014 | 0.053 | 0.000 | 0.000 | 0 |

Source: own computations in Matlab

**Appendix 3**

Posterior Summary Estimates for explaining stress and anxiety scores according to labour status

| Parameter | Stress | | | | | Anxiety | | | | |
| --- | --- | --- | --- | --- | --- | --- | --- | --- | --- | --- |
|  | Mean | Standard deviation | 25% | 75% | CUSUM | Mean | Standard deviation | 25% | 75% | CUSUM |
| $\beta_{0}(sample)$ | 47.831 | 26.686 | 54.282 | 62.433 | 0.17 | 27.387 | 19.021 | 12.666 | 39.392 | 0.232 |
| $\beta\left( sample \right):environment$ | 1.399 | 3.385 | -0.560 | 4.366 | 0.364 | 0.229 | 2.076 | 0.457 | 0.469 | 0.236 |
| $\beta\left( sample \right):age$ | -0.465 | 2.307 | -1.794 | 0.352 | 0.143 | -0.810 | 2.056 | -1.205 | -1.191 | 0.245 |
| $\beta\left( sample \right):gender$ | -0.734 | 2.326 | -1.323 | 0.844 | 0.199 | 2.831 | 2.737 | 2.888 | 4.016 | 0.301 |
| $\beta\left( sample \right):specialization$ | 1.028 | 5.136 | -2.778 | 3.499 | 0.366 | 0.050 | 1.940 | 0.122 | 0.133 | 0.224 |
| $\sigma_{sample}^{2}$ | 196.252 | 92.777 | 240.493 | 240.493 | 0 | 62.171 | 42.800 | 1.871 | 93.018 | 0.288 |
| $\mu_{\beta_{0}}$ | 2.463 | 3.263 | 0.249 | 4.745 | 0.508 | 3.048 | 3.256 | 0.841 | 5.280 | 0.5 |
| $\mu_{environment}$ | 1.302 | 3.122 | -0.755 | 3.404 | 0.368 | -0.201 | 2.989 | -2.295 | 1.847 | 0.323 |
| $\mu_{age}$ | -0.041 | 3.189 | -2.249 | 2.259 | 0.227 | -0.120 | 3.025 | -2.212 | 1.926 | 0.317 |
| $\mu_{gender}$ | -0.802 | 3.047 | -2.958 | 1.332 | 0.252 | 0.425 | 3.088 | -1.668 | 2.462 | 0.34 |
| $\mu_{specialization}$ | -0.772 | 3.083 | -2.874 | 1.266 | 0.302 | -0.084 | 2.999 | -2.068 | 1.950 | 0.326 |
| $\tau_{\beta_{0}}$ | 911.035 | 618.359 | 550.168 | 1077.120 | 0.503 | 223.729 | 167.747 | 126.163 | 268.914 | 0.504 |
| $\tau_{\beta_{0}: \beta_{environment}}$ | -18.484 | 73.274 | -47.341 | 14.972 | 0.387 | 4.266 | 24.164 | -7.173 | 14.293 | 0.332 |
| $\tau_{\beta_{0}: \beta_{age}}$ | 3.239 | 61.140 | -27.216 | 32.108 | 0.235 | -6.498 | 24.372 | -16.281 | 5.127 | 0.343 |
| $\tau_{\beta_{0}: \beta_{gender}}$ | 20.673 | 64.900 | -12.603 | 49.162 | 0.267 | 21.477 | 28.058 | 6.007 | 30.394 | 0.39 |
| $\tau_{\beta_{0}: \beta_{specialization}}$ | 13.463 | 66.433 | -18.385 | 41.069 | 0.325 | 1.324 | 24.316 | -9.391 | 11.473 | 0.348 |
| $\tau_{\beta_{environment}}$ | 8.737 | 8.561 | 4.434 | 10.375 | 0.480 | 3.284 | 3.566 | 1.379 | 3.821 | 0.442 |
| $\tau_{\beta_{envrionment}:\beta_{age}}$ | 0.121 | 4.446 | -1.217 | 1.413 | 0.444 | -0.119 | 2.373 | -0.813 | 0.673 | 0.462 |
| $\tau_{\beta_{environment}:\beta_{gender}}$ | -2.352 | 4.831 | -3.777 | -0.474 | 0.458 | 0.440 | 3.185 | -0.744 | 1.395 | 0.409 |
| $\tau_{\beta_{environment}:\beta_{specialization}}$ | -3.141 | 5.263 | -4.490 | -1.006 | 0.467 | 0.027 | 2.336 | -0.690 | 0.733 | 0.451 |
| $\tau_{\beta_{age}}$ | 4.301 | 4.792 | 1.510 | 5.156 | 0.375 | 3.368 | 3.582 | 1.434 | 3.859 | 0.443 |
| $\tau_{\beta_{age}:\beta_{gender}}$ | -0.033 | 3.894 | -1.241 | 1.102 | 0.374 | -0.592 | 3.471 | -1.504 | 0.638 | 0.392 |
| $\tau_{\beta_{age}:\beta_{specialization}}$ | 0.001 | 3.885 | -1.262 | 1.161 | 0.404 | -0.042 | 2.284 | -0.732 | 0.689 | 0.455 |
| $\tau_{\beta_{gender}}$ | 5.418 | 5.977 | 2.115 | 6.307 | 0.403 | 5.333 | 6.153 | 1.924 | 6.355 | 0.38 |
| $\tau_{\beta_{gender}:\beta_{specialization}}$ | 1.430 | 4.345 | -0.018 | 2.719 | 0.436 | 0.178 | 3.152 | -0.898 | 1.145 | 0.406 |
| $\tau_{\beta_{specialization}}$ | 6.233 | 6.339 | 2.851 | 7.252 | 0.453 | 3.192 | 3.416 | 1.368 | 3.656 | 0.457 |
| $\alpha$ | 3.597 | 2.672 | 1.676 | 4.744 | 0.502 | 2.110 | 2.310 | 0.571 | 2.812 | 0.499 |
| $\beta_{0}(employed)$ | 54.282 | 0.000 | 54.282 | 54.282 | 0 | 39.385 | 0.011 | 39.374 | 39.397 | 0 |
| $\beta_{0}(unemployed)$ | 62.508 | 0.000 | 62.508 | 62.508 | 0 | 39.385 | 0.011 | 39.374 | 39.397 | 0 |
| $\beta_{environment}(employed)$ | 4.366 | 0.000 | 4.366 | 4.366 | 0 | 0.464 | 0.005 | 0.457 | 0.468 | 0 |
| $\beta_{environment}(nonemployed)$ | -3.337 | 0.000 | -3.337 | -3.337 | 0 | 0.464 | 0.005 | 0.457 | 0.468 | 0 |
| $\beta_{age}(employed)$ | 0.352 | 0.000 | 0.352 | 0.352 | 0 | -1.199 | 0.007 | -1.203 | -1.191 | 0 |
| $\beta_{age}(nonemployed)$ | -0.049 | 0.000 | -0.049 | -0.049 | 0 | -1.199 | 0.007 | -1.203 | -1.191 | 0 |
| $\beta_{gender}(employed)$ | -1.323 | 0.000 | -1.323 | -1.323 | 0 | 4.015 | 0.002 | 4.014 | 4.018 | 0 |
| $\beta_{gender}(nonemployed)$ | 2.035 | 0.000 | 2.035 | 2.035 | 0 | 4.015 | 0.002 | 4.014 | 4.018 | 0 |
| $\beta_{specialization}(employed)$ | -2.778 | 0.000 | -2.778 | -2.778 | 0 | 0.131 | 0.004 | 0.130 | 0.133 | 0.001 |
| $\beta_{specialization}(nonemployed)$ | 2.362 | 0.000 | 2.362 | 2.362 | 0 | 0.131 | 0.004 | 0.130 | 0.133 | 0.001 |
| $s^{2}(employed)$ | 240.493 | 0.000 | 240.493 | 240.493 | 0 | 92.427 | 0.931 | 91.329 | 93.340 | 0 |
| $s^{2}(nonemployed)$ | 240.493 | 0.000 | 240.493 | 240.493 | 0 | 92.427 | 0.931 | 91.329 | 93.340 | 0 |
| ICC | 0.799 | 0.123 | 0.711 | 0.872 | 0.508 | 0.769 | 0.184 | 0.620 | 0.989 | 0.489 |
| Reliability ($\beta_{R_{0}})$ | 0.999 | 0.001 | 0.998 | 0.999 | 0.508 | 0.998 | 0.002 | 0.997 | 1.000 | 0.489 |
| Reliability ($\beta_{R_{environment}})$ | 0.866 | 0.093 | 0.802 | 0.934 | 0.49 | 0.874 | 0.118 | 0.790 | 0.995 | 0.471 |
| Reliability ($\beta_{R_{age}})$ | 0.736 | 0.188 | 0.585 | 0.896 | 0.416 | 0.878 | 0.114 | 0.797 | 0.995 | 0.472 |
| Reliability ($\beta_{R_{gender}})$ | 0.782 | 0.156 | 0.665 | 0.911 | 0.428 | 0.904 | 0.100 | 0.842 | 0.996 | 0.453 |
| Reliability ($\beta_{R_{specialization}})$ | 0.815 | 0.130 | 0.720 | 0.920 | 0.462 | 0.874 | 0.116 | 0.790 | 0.994 | 0.482 |
| $\beta_{s^{2}}(acceptance rate)$ | 0.002 | 0.016 | 0 | 0 | 0 | 0.008 | 0.091 | 0 | 0 | 0 |

Source: own computations in Matlab

**Appendix 4**

Posterior Summary Estimates for explaining stress and anxiety scores according to specialization

| Parameter | Stress | | | | | Anxiety | | | | |
| --- | --- | --- | --- | --- | --- | --- | --- | --- | --- | --- |
|  | Mean | Standard deviation | 25% | 75% | CUSUM | Mean | Standard deviation | 25% | 75% | CUSUM |
| $\beta_{0}(sample)$ | 59.324 | 29.903 | 65.898 | 72.114 | 0.093 | 36.437 | 4.296 | 34.511 | 39.251 | 0.47 |
| $\beta\left( sample \right):employed$ | 7.717 | 4.274 | 7.511 | 10.103 | 0.002 | -0.234 | 2.319 | -1.926 | 0.372 | 0.417 |
| $\beta\left( sample \right):environment$ | -4.733 | 3.620 | -6.957 | -1.443 | 0.071 | -0.653 | 3.295 | -3.138 | 2.621 | 0.493 |
| $\beta\left( sample \right):age$ | -0.605 | 2.147 | -1.340 | -0.251 | 0.133 | 0.286 | 2.798 | -0.974 | 1.663 | 0.439 |
| $\beta\left( sample \right):gender$ | 1.176 | 2.237 | -0.775 | 2.405 | 0.031 | 1.578 | 3.567 | -1.044 | 3.733 | 0.488 |
| $\sigma_{sample}^{2}$ | 192.355 | 88.220 | 233.181 | 233.181 | 0 | 77.974 | 2.316 | 78.240 | 78.240 | 0.044 |
| $\mu_{\beta_{0}}$ | 1.606 | 3.189 | -0.575 | 3.685 | 0.508 | 5.058 | 3.489 | 2.718 | 7.297 | 0.483 |
| $\mu_{employed}$ | 0.441 | 3.178 | -1.701 | 2.604 | 0.307 | -1.560 | 2.889 | -3.498 | 0.368 | 0.314 |
| $\mu_{environment}$ | -0.309 | 3.130 | -2.443 | 1.828 | 0.32 | 2.279 | 2.801 | 0.402 | 4.230 | 0.299 |
| $\mu_{age}$ | 0.040 | 3.089 | -2.058 | 2.119 | 0.318 | -0.550 | 2.568 | -2.253 | 1.203 | 0.243 |
| $\mu_{gender}$ | -0.110 | 3.122 | -2.195 | 2.029 | 0.317 | 0.011 | 2.537 | -1.684 | 1.682 | 0.265 |
| $\tau_{\beta_{0}}$ | 831.565 | 570.759 | 485.917 | 976.074 | 0.506 | 267.896 | 173.840 | 155.263 | 327.740 | 0.478 |
| $\tau_{\beta_{0}: \beta_{employed}}$ | 113.497 | 90.864 | 57.988 | 140.002 | 0.441 | 19.880 | 35.360 | 0.339 | 34.958 | 0.344 |
| $\tau_{\beta_{0}: \beta_{environment}}$ | -78.094 | 68.970 | -98.877 | -36.326 | 0.405 | -23.846 | -37.924 | -37.808 | -3.391 | 0.332 |
| $\tau_{\beta_{0}: \beta_{age}}$ | -3.516 | 47.700 | -23.592 | 17.853 | 0.338 | 1.461 | 30.170 | -12.576 | 14.842 | 0.248 |
| $\tau_{\beta_{0}: \beta_{gender}}$ | 29.230 | 53.092 | 2.181 | 47.625 | 0.34 | -4.322 | 28.549 | -16.903 | 10.371 | 0.271 |
| $\tau_{\beta_{employed}}$ | 18.819 | 17.805 | 8.030 | 23.581 | 0.389 | 7.672 | 7.394 | 3.299 | 9.419 | 0.424 |
| $\tau_{\beta_{employed}:\beta_{environment}}$ | -10.739 | 10.599 | -14.209 | -4.122 | 0.379 | -3.307 | 6.098 | -4.894 | -0.614 | 0.4 |
| $\tau_{\beta_{employed}:\beta_{age}}$ | -0.444 | 6.930 | -3.046 | 2.335 | 0.352 | 0.091 | 3.946 | -1.140 | 1.301 | 0.385 |
| $\tau_{\beta_{employed}:\beta_{gender}}$ | 3.989 | 7.944 | 0.232 | 6.323 | 0.347 | -0.158 | 3.667 | -1.342 | 1.108 | 0.374 |
| $\tau_{\beta_{environment}}$ | 10.605 | 10.819 | 3.798 | 13.524 | 0.347 | 7.477 | 9.173 | 2.872 | 8.833 | 0.36 |
| $\tau_{\beta_{environment}:\beta_{age}}$ | 0.336 | 5.038 | -1.581 | 2.114 | 0.357 | -0.336 | 4.111 | -1.468 | 1.065 | 0.366 |
| $\tau_{\beta_{environment}:\beta_{gender}}$ | -2.827 | 5.656 | -4.400 | -0.013 | 0.344 | 0.266 | 4.015 | -1.087 | 1.369 | 0.394 |
| $\tau_{\beta_{age}}$ | 3.282 | 3.768 | 1.393 | 3.708 | 0.448 | 3.703 | 4.475 | 1.312 | 4.179 | 0.375 |
| $\tau_{\beta_{age}:\beta_{gender}}$ | -0.147 | 3.054 | -0.896 | 0.735 | 0.419 | 0.006 | 2.933 | -0.848 | 0.848 | 0.409 |
| $\tau_{\beta_{gender}}$ | 4.386 | 5.586 | 1.566 | 5.177 | 0.403 | 3.677 | 4.234 | 1.327 | 4.339 | 0.509 |
| $\alpha$ | 2.495 | 2.694 | 0.661 | 3.316 | 0.504 | 5.391 | 3.706 | 2.720 | 7.158 | 0 |
| $\beta_{0}(spec1)$ | 72.114 | 0 | 72.114 | 72.114 | 0 | 34.502 | 0 | 34.502 | 34.502 | 0 |
| $\beta_{0}(spec2)$ | 72.114 | 0 | 72.114 | 72.114 | 0 | 34.388 | 0.953 | 34.502 | 34.502 | 0 |
| $\beta_{0}(spec3)$ | 72.114 | 0 | 72.114 | 72.114 | 0 | 34.502 | 0 | 34.502 | 34.502 | 0 |
| $\beta_{0}(spec4)$ | 72.114 | 0 | 72.114 | 72.114 | 0 | 27.481 | 2.741 | 26.442 | 26.442 | 0.02 |
| $\beta_{0}(spec5)$ | 72.114 | 0 | 72.114 | 72.114 | 0 | 33.100 | 3.666 | 34.511 | 34.511 | 0.019 |
| $\beta_{employed}(spec1)$ | 10.103 | 0 | 10.103 | 10.103 | 0 | 3.511 | 0 | 3.511 | 3.511 | 0 |
| $\beta_{employed}(spec2)$ | 10.103 | 0 | 10.103 | 10.103 | 0 | 3.427 | 0.697 | 3.511 | 3.511 | 0 |
| $\beta_{employed}(spec3)$ | 10.103 | 0 | 10.103 | 10.103 | 0 | 3.511 | 0 | 3.511 | 3.511 | 0 |
| $\beta_{employed}(spec4)$ | 10.103 | 0 | 10.103 | 10.103 | 0 | -1.669 | 1.908 | -2.386 | -2.386 | 0.022 |
| $\beta_{employed}(spec5)$ | 10.103 | 0 | 10.103 | 10.103 | 0 | -0.791 | 1.080 | -0.333 | -0.333 | 0 |
| $\beta_{environment}(spec1)$ | -6.957 | 0 | -6.957 | -6.957 | 0 | -2.187 | 0 | -2.187 | -2.187 | 0 |
| $\beta_{environment}(spec2)$ | -6.957 | 0 | -6.957 | -6.957 | 0 | -2.119 | 0.568 | -2.187 | -2.187 | 0 |
| $\beta_{environment}(spec3)$ | -6.957 | 0 | -6.957 | -6.957 | 0 | -2.187 | 0 | -2.187 | -2.187 | 0 |
| $\beta_{environment}(spec4)$ | -6.957 | 0 | -6.957 | -6.957 | 0 | 2.032 | 1.562 | 2.621 | 2.621 | 0.019 |
| $\beta_{environment}(spec5)$ | -6.957 | 0 | -6.957 | -6.957 | 0 | 0.932 | 1.294 | 0.757 | 0.757 | 0.06 |
| $\beta_{age}(spec1)$ | -0.251 | 0 | -0.251 | 0.251 | 0 | -0.396 | 0 | -0.396 | -0.396 | 0 |
| $\beta_{age}(spec2)$ | -0.251 | 0 | -0.251 | 0.251 | 0 | -0.400 | 0.030 | -0.396 | -0.396 | 0 |
| $\beta_{age}(spec3)$ | -0.251 | 0 | -0.251 | 0.251 | 0 | -0.396 | 0 | -0.396 | -0.396 | 0 |
| $\beta_{age}(spec4)$ | -0.251 | 0 | -0.251 | 0.251 | 0 | -0.617 | 0.088 | -0.650 | -0.650 | 0.013 |
| $\beta_{age}(spec5)$ | -0.251 | 0 | -0.251 | 0.251 | 0 | -0.357 | 0.184 | -0.261 | -0.261 | 0.023 |
| $\beta_{gender}(spec1)$ | 2.405 | 0 | 2.405 | 2.405 | 0 | -0.228 | 0 | -0.228 | -0.228 | 0 |
| $\beta_{gender}(spec2)$ | 2.405 | 0 | 2.405 | 2.405 | 0 | -0.229 | 0.006 | -0.228 | -0.228 | 0 |
| $\beta_{gender}(spec3)$ | 2.405 | 0 | 2.405 | 2.405 | 0 | -0.228 | 0 | -0.228 | -0.228 | 0 |
| $\beta_{gender}(spec4)$ | 2.405 | 0 | 2.405 | 2.405 | 0 | -0.265 | 0.247 | -0.275 | -0.275 | 0 |
| $\beta_{gender}(spec5)$ | 2.405 | 0 | 2.405 | 2.405 | 0 | -0.707 | 1.043 | -1.044 | -0.730 | 0.01 |
| $s^{2}(spec1)$ | 233.181 | 0 | 233.181 | 233.181 | 0 | 78.24 |  | 78.24 | 78.24 | 0 |
| $s^{2}(spec2)$ | 233.181 | 0 | 233.181 | 233.181 | 0 | 78.207 | 0.269 | 78.24 | 78.24 | 0 |
| $s^{2}(spec3)$ | 233.181 | 0 | 233.181 | 233.181 | 0 | 78.24 | 0 | 78.24 | 78.24 | 0 |
| $s^{2}(spec4)$ | 233.181 | 0 | 233.181 | 233.181 | 0 | 76.250 | 0.760 | 75.960 | 75.960 | 0 |
| $s^{2}(spec5)$ | 233.181 | 0 | 233.181 | 233.181 | 0 | 77.765 | 0.926 | 78.240 | 78.240 | 0.048 |
| ICC | 0.786 | 0.130 | 0.691 | 0.868 | 0.51 | 0.732 | 0.103 | 0.665 | 0.808 | 0.478 |
| Reliability ($\beta_{R_{0}})$ | 0.990 | 0.007 | 0.986 | 0.995 | 0.509 | 0.987 | 0.007 | 0.984 | 0.992 | 0.477 |
| Reliability ($\beta_{R_{employed}})$ | 0.747 | 0.169 | 0.639 | 0.860 | 0.443 | 0.731 | 0.110 | 0.652 | 0.818 | 0.423 |
| Reliability ($\beta_{R_{environment}})$ | 0.655 | 0.215 | 0.498 | 0.795 | 0.415 | 0.714 | 0.123 | 0.626 | 0.810 | 0.373 |
| Reliability ($\beta_{R_{age}})$ | 0.477 | 0.263 | 0.281 | 0.576 | 0.462 | 0.585 | 0.150 | 0.468 | 0.695 | 0.393 |
| Reliability ($\beta_{R_{gender}})$ | 0.511 | 0.260 | 0.304 | 0.653 | 0.422 | 0.590 | 0.148 | 0.471 | 0.702 | 0.409 |
| $\beta_{s^{2}}(acceptance rate)$ | 0.002 | 0.023 | 0 | 0 | 0 | 0.010 | 0.011 | 0 | 0.012 | 0.181 |

Source: own computations in Matlab
